# Does intermittent pneumatic compression reduce the risk of post stroke deep vein thrombosis? The CLOTS 3 trial: statistical analysis plan

**DOI:** 10.1186/1745-6215-14-66

**Published:** 2013-03-06

**Authors:** Martin Dennis, Peter Sandercock, Gordon Murray, John Forbes

**Affiliations:** 1 Division of Clinical Neurosciences, The University of Edinburgh, Western General Hospital, Crewe Road, Edinburgh, EH4 2XU, UK; 2 Centre for Population Health Sciences, The University of Edinburgh, College of Medicine and Veterinary Medicine, Teviot Place, Edinburgh, EH8 9AG, UK

**Keywords:** Stroke, Deep vein thrombosis, Prevention, Intermittent pneumatic compression, Statistical analysis plan

## Abstract

**Background:**

Venous thromboembolism (VTE) is a common and important complication of stroke. The CLOTS 3 trial aims to determine whether, compared with best medical care, best medical care plus intermittent pneumatic compression (IPC) in immobile stroke patients reduces the risk of proximal deep vein thrombosis (DVT).

**Methods/Design:**

The CLOTS 3 trial is a multicenter, parallel group trial with centralized randomization (minimization) to ensure allocation concealment. The protocol has been published (*Trials* 2012, **13**:26) and is available in full at: http://www.clotstrial.com. Between December 2008 and September 2012, 105 centers in the UK recruited 2,876 immobile stroke patients within the first 3 days of their hospital admission. Patients were allocated to best medical care or best medical care plus IPC. Ultrasonographers performed a compression Doppler ultrasound scan to detect DVT in each treatment group at 7 to 10 days and 25 to 30 days. The primary outcome cluster includes symptomatic or asymptomatic DVT in the popliteal or femoral veins detected on either scan. Patients will be followed up by postal or telephone questionnaire at 6 months from randomization to detect later symptomatic DVT and pulmonary embolism (PE), and to measure functional outcome (Oxford Handicap Scale) and quality of life (EQ-5D-3L). The ultrasonographers performing the scans are blinded to treatment allocation, whereas the patients and caregivers are not. The trial has more than 90% power to detect a 4% absolute difference (12% versus 8%) in risk of the primary outcome and includes a health economic analysis.

Follow-up will be completed in April 2013 and the results reported in May 2013. In this update, we describe the statistical analysis plan.

**Trial registration:**

ISRCTN: ISRCTN93529999

## Update

### Introduction

A description of the CLOTS 3 trial protocol has already been published [1] and that article contained a brief description of planned analyses. However, to avoid criticism that our analyses are *post hoc* or data driven, we have published this more detailed statistical analysis plan in advance of closing the database and performing analyses. None of the authors of this analysis plan have had access to any interim analyses which split the patients by treatment group. The trial statistician who prepared the interim analyses for the independent data monitoring committee (DMC) was not involved in the writing of this analysis plan.

The overall analysis strategy is similar to that reported in the published CLOTS 1 and 2 trials [2,3].

Before describing the planned analyses, the key methodological features of the CLOTS 3 trial will be presented. It is a multicenter, parallel group trial with centralized randomization (minimization) and 1:1 allocation to ensure allocation concealment.

### Primary research question in the CLOTS 3 trial

Does early and routine application of intermittent pneumatic compression (IPC), in addition to routine care, reduce the risk of above knee deep vein thrombosis (DVT) in the weeks following an acute stroke?

### Minimization algorithm

A minimization program is used to achieve optimum balance for the following important prognostic factors:

1. Delay since stroke onset (day 0 or 1 versus day 2 to 7).

2. Stroke severity (using a validated prognostic model [4], which includes the factors: age, pre-stroke dependency in activities of daily living (ADL), living with another person prior to stroke, able to talk and orientated in time, place and person, and able to lift both arms to horizontal position against gravity).

3. Severity of leg paresis (able, or not, to lift leg off the bed).

4. Use of heparin, warfarin or thrombolysis (recombinant tissue plasminogen activator (rtPA)) at time of enrolment.

### Primary outcome

The primary outcome is the presence of definite or probable symptomatic or asymptomatic DVT in the popliteal or femoral veins detected on compression Doppler ultrasound scan, or any symptomatic DVT in the popliteal or femoral veins confirmed on compression Doppler ultrasound, contrast venography or MRI direct thrombus imaging within 30 days of randomization.

### Secondary outcomes

The secondary outcomes are:

1. Presence of definite or probable DVT in the popliteal or femoral veins detected on a screening compression Doppler ultrasound scan, which was not clinically suspected before the scan.

2. Definite (excluding probable DVTs) symptomatic or asymptomatic DVT in the popliteal or femoral veins detected on compression Doppler ultrasound scan, contrast venography or MRI direct thrombus imaging within 30 days of randomization.

3. Any definite or probable symptomatic or asymptomatic DVT (including DVTs, which only involve the calf veins).

4. Confirmed fatal or non-fatal pulmonary embolism (PE).

5. Adherence to allocated treatment.

6. Adverse events related to IPC within 30 days of randomization.

7. Patient death within 30 days of randomization.

#### Secondary outcomes at 6 months

The secondary outcomes at 6 months are:

1. Any confirmed symptomatic or asymptomatic DVT or PE occurring between randomization and final follow-up.

2. Any symptomatic DVT or PE occurring between randomization and final follow-up.

3. Place of residence.

4. Post DVT syndrome.

5. Disability (Oxford Handicap Scale (OHS) [5], simple questions [6]).

6. Health-related quality of life (EQ-5D-3L, EuroQol, Rotterdam, The Netherlands [7]).

7. Death from any cause.

The later effects of DVT/PE (for example breathlessness, leg pain, leg swelling and poor stroke recovery) or the adverse events related to IPC (falls with injury, fractures, skin ulceration, amputation and loss of mobility) may be diverse; therefore, a measure of overall health-related quality of life is included.

### Adverse events

The adverse events are:

1. Any damage to the skin of the legs including necrosis and ulcers occurring within 30 days of enrollment.

2. Any reasons for prematurely stopping the IPC.

3. Any fall associated with significant injury occurring within 30 days of enrollment (when IPC might still be applied). These are injuries that require investigation, specific treatment, prolong hospitalization, or lead to temporary or permanent disability, or death.

4. Fractures.

### Follow-up

Patients will have a compression Doppler ultrasound scan examination of the veins in both legs between day 7 and 10, and between day 25 and 30. This examination will document thrombus in the calf, popliteal and femoral veins separately.

The data collected at discharge will include:

1. Use of heparin, warfarin and antiplatelet drugs during admission, to monitor the components of routine care and to ensure equal use in the two treatment arms. The indication for the use of anticoagulants is recorded.

2. Use of full length or below knee graduated compression stockings, to monitor the components of routine care and to ensure equal use in the two treatment arms.

3. Timing of initiation of IPC, adherence to treatment allocation and use of IPC. If IPC is prematurely stopped, the reason is recorded.

4. Any clinical DVT or PE.

5. Any treatment complications, in particular skin problems with legs and falls resulting in injuries, occurring within the first 30 days after enrolment.

### Follow-up at 6 months

For patients who have been discharged, outcome will be assessed via a postal or telephone questionnaire to establish:

1. That the patient is alive; or, if applicable, the date and cause of death.

2. Whether the patient has suffered a DVT or PE since discharge.

3. The place of residence (own home, with relatives, residential care or nursing home), as a guide to resource use.

4. Functional status measured by the degree of functional impairment on the OHS, simple questions and health-related quality of life using EQ-5D-3L.

5. Current antithrombotic medication regimen.

6. Presence of symptoms which might reflect post DVT syndrome (for example leg swelling and ulcers).

### Ethical Approval

The protocol was approved by the Multicentre Research Ethics committees (in Scotland and England). Informed consent was obtained from all participants, or if they lack capacity, from a personal legal representative.

### Statistical analysis plan

All analyses will be based on intention-to-treat. We will analyze patients in the groups they were randomized to, regardless of treatment received. We will strive to obtain full follow-up data on every patient to allow a full intention-to-treat analysis, but inevitably some patients will be lost to follow-up. We will exclude these patients from the analyses with no data and undertake sensitivity analyses to examine the effect of exclusions on the conclusions. For binary outcomes (for example occurrence, or not, of a primary or secondary outcome), outcomes will be presented as odds ratios and 95% confidence intervals (CIs), adjusted using logistic regression for the factors used in the minimization algorithm. We will calculate absolute reductions in risk from these values along with 95% CIs.

### Primary analysis

The primary analysis will aim to establish whether a policy of early routine application of IPC to immobile stroke patients, within the first 3 days of hospital admission and within 7 days of the stroke onset, reduces the frequency of the primary outcome (symptomatic or asymptomatic DVT within 30 days). This will compare the occurrence of the primary outcome in the two arms of the trial. Patients in each arm will be categorized as:

1. Alive no DVT (no primary outcome).

2. Alive with DVT (primary outcome).

3. Died prior to any primary outcome.

4. Missing.

The primary analysis will compare IPC with no IPC for the occurrence of DVT (death and missing excluded), adjusted for the variables included in the minimization algorithm by logistic regression. The treatment effect will be presented as an odds ratio with 95% CIs. We will give a *P* value and change in −2 log-likelihood. We will also present unadjusted estimates.

In addition, we will perform a sensitivity analysis comparing IPC with no IPC for the occurrence of DVT (with death counted as DVT and missing as no DVT).

### Secondary analyses

#### Minimizing observer bias

Although we strive to blind the radiographer to the treatment allocation, our primary outcome may be prone to observer bias. For example, if a patient presents clinical signs of DVT, the ward staff may push harder to obtain the routine compression Duplex ultrasound scan. We will therefore repeat the primary analysis but exclude patients with a primary outcome in whom a DVT was suspected before the ultrasound scan.

#### Safety

There are a number of adverse events that might occur with IPC use. For example IPC might increase the risk of PE by squeezing the leg in patients who have already developed a DVT; patients who attempt to mobilize with the IPC sleeves attached might fall and suffer injury; or the sleeves or intermittent pressure may cause patients to develop skin breaks on their legs. Any of these events could be fatal.

We will therefore compare the occurrence of the following events within 30 days of randomization in the IPC and no IPC groups: death from any cause, confirmed PE (fatal or non-fatal), skin breaks, fall with injury and fractures.

#### Subtypes of venous thrombembolism

It is possible that IPC may reduce the frequency of one type of venous thrombembolism (VTE) (for example below knee DVT) whilst increase the risk of another (for example PE). We will therefore repeat our primary analysis and subcategorize VTE as:

1. Any DVT (symptomatic or asymptomatic DVT, affecting calf only and/or proximal veins occurring within 30 days or by second compression Doppler ultrasound scan).

2. Only symptomatic DVT (defined as associated with leg swelling, pain or warmth) whether affecting proximal veins or calf only occurring within 30 days.

3. Confirmed fatal or non-fatal PE occurring within 30 days.

### Subgroup analyses

Subgroup analyses will be performed to determine whether certain types of patients might gain greater or less benefit from IPC. We will estimate the effect of treatment allocation on the primary outcome subdivided by key baseline variables and adjusted for the other factors included in the minimization algorithm. Subgroup analyses will be performed by observing the change in log-likelihood when the interaction between the treatment and the subgroup is added into a logistic regression model. We will determine whether there is significant heterogeneity between these subgroups.

#### Analysis of impact of delay in applying IPC

There is evidence that DVTs may develop within the first 3 days after a stroke [8]. There is often a delay in presenting with a stroke and there will always be a delay in applying VTE prophylaxis, since the patients need to be assessed and the treatment started. In addition, in a randomized trial there may be additional delays owing to the time taken to obtain consent and enroll the patients. To reduce the possible impact of this delay, we encourage collaborators to enroll patients as early as possible after admission and stipulated that at least 75% of patients are enrolled within day 0 (day of stroke) and day 2.

We hypothesize that IPC will reduce the frequency of the primary outcome to a greater extent among patients enrolled early compared to later. We will therefore examine the effect of treatment among patients enrolled early versus later. We will also assess the effect of delay by applying two different definitions of delay (measured in days from stroke onset to enrolment): day 0 to 1 versus day 2 to 7 (the split on which the minimization algorithm is based), and day 0 to 2 versus day 3 to 7 (as stipulated in the sample size estimates).

Other preplanned subgroup analyses, described in our original protocol, include analyses of the effect of treatment on the primary outcome subdivided by: paralysis of leg (able to lift both legs versus unable to lift both legs) at randomization and probability of survival free of dependency (OHS <3) based on the predictive model used in minimization.

We hypothesize that patients with predicted worse outcomes have higher rates of DVT because of more prolonged immobility and the co-occurrence of infections. Also, this group may have better adherence to the IPC, since these patients are not as able to express a wish to have them removed.

#### Is IPC more effective in patients at higher risk of DVT?

It seems likely that patients at higher risk of DVT might gain greater benefit from IPC than those at lower risk. In the CLOTS 1 and 2 trials, we showed that immobile stroke patients with the following features had a greater risk of proximal DVT [9]: dependent in ADL prior to stroke, prior history of DVT, unable to lift both arms and unable to lift both legs. We will undertake subgroup analyses of the effect of allocation to IPC on the primary outcome among patients with and without at least one of these factors at baseline.

#### Is IPC more effective in patients in whom anticoagulation is not given or advisable?

In some health systems anticoagulation is widely used for the prophylaxis of DVT in patients with ischaemic stroke despite the lack of evidence that it improves overall outcome. Consequently, IPC and other forms of compression have often been used in patients with hemorrhagic stroke. We will therefore carry out the following subgroup analyses of patients on anticoagulants (as defined in our protocol [1]) versus not at baseline and with confirmed hemorrhagic versus ischaemic or unknown pathological type of stroke.

#### Duration and intensity of treatment with IPC

IPC has usually been used for a relatively short time on perioperative patients and patients in high dependency units. In the CLOTS 3 trial, we aimed to maintain the IPC treatment for up to 30 days, if the patient remained in hospital and was still immobile. However, for a variety of reasons, the IPC was often removed earlier than this or was applied non-continuously. In CLOTS 1 and 2 trials, 79% of proximal DVTs were detected on the first compression Doppler ultrasound scan [10]. The risk of DVT seemed to be highest early on and therefore any prophylaxis might be more effective during this period.

We will therefore carry out additional analyses (including all those subgroups identified in the primary outcome) of the effect of allocation to IPC on the primary outcome occurring within 14 days of randomization, rather than 30 days. This will, to some extent, reduce the impact of patients who only required IPC for a few days before becoming mobile or only adhered to IPC for a short time. Also, it will reflect the practice in many places where acute hospital admission for stroke is short and prophylaxis is only applied for the first few days. However, it is also possible that prophylaxis for the first 7, 14 or 30 days may simply defer the onset of DVT. We will therefore analyze whether there is a difference in frequency of any symptomatic and/or asymptomatic DVT or PE within 6 months between patients allocated to IPC, or not.

During the CLOTS 3 trial, it was noted that adherence to the IPC was suboptimal. The manufacturer responded to this information by introducing a modified IPC sleeve, the Kendall™ SCD Sequential Compression Comfort Sleeves, which aimed to improve acceptability and adherence. We switched to using this new sleeve on 17th October 2011. Therefore, the first 1679 (approximately 59%) patients enrolled in the CLOTS 3 trial were allocated to the original sleeve, or not; and the subsequent patients to the Comfort sleeve, or not. We will therefore examine the effect of the original and Comfort sleeves separately. We will also compare the adherence to the sleeves, which will be based on a non-randomized comparison.

#### If primary analyses fail to demonstrate a reduction of DVT with IPC, is this likely to be due to poor adherence?

If the primary or secondary analyses do not provide evidence that IPC reduces the risk of DVT, we will perform further analyses to establish the extent to which poor adherence to the IPC might explain this lack of effect.

We will build a statistical model based on baseline factors in the IPC group to predict the degree of adherence to IPC. We will then use this model to dichotomize patients in both groups at baseline into patients likely to have high and low adherence. We will then examine whether the effect is greater in patients with a likelihood of better adherence to IPC.

### Overall benefit

Although the primary aim of the CLOTS 3 trial is to establish whether IPC reduces the risk of post stroke DVT, it is also important to determine whether it improves overall outcome [11]. However, the CLOTS 3 trial was not powered to establish whether IPC influences longer-term outcome. We will, however, compare the following longer-term outcomes in patients allocated IPC, or not, adjusted for baseline factors in the same way as the primary analyses:

1. Survival to 6 months (Cox proportional hazards model).

2. Living circumstances at final follow-up (living at home versus living in an institution/still in hospital).

3. Death, dependent, independent but with problems or no problems (assessed by simple questions).

4. OHS at final follow-up, analyzed in two ways: dichotomized in OHS 0 to 2 versus 3 to 6 (by logistic regression) and as an ordinal scale (by ordinal regression).

5. Health-related quality of life (measured by EQ-5D-3L).

The utility based on the EQ-5D-3L will be compared by t-tests if the data are normally distributed and use an appropriate nonparametric test otherwise. The EQ-5D-3L data will be analyzed in two ways: one excluding the patients who died and for whom we do not have 6 month EQ-5D-3L; and the other which includes the patients who have died, giving them a utility of 0, but excluding those with missing data.

### Economic analyses

Economic analysis of trial treatment effects will involve a within-trial evaluation of cost effectiveness integrated into a decision-analytic model of longer run costs and health effects. The within-trial analysis will be conducted on an intention-to-treat basis. The primary health endpoints will be survival times adjusted for quality of life. A standard multiplicative model will be used to estimate quality adjusted life years (QALYs) by the area under linear interpolation of the EQ-5D-3L index trajectory for each individual patient using survival times, the EQ-5D-3L index score at 6 months and a modeled baseline EQ-5D-3L index score. We will assess robustness using probabilistic sensitivity analysis of the parameters used to generate the short-run QALY estimates.

A NHS perspective will be adopted for assessing resource use and costs. Patient-specific hospital resource use will be measured using the duration of stay for the index episode following randomization. The net direct medical cost will include the hospital stay, converted into cost estimates using NHS *per diem* hospital costs, a cost estimate of IPC capital/equipment (and staffing implications) and the averted costs arising from the effects of IPC on expected DVT/PE incidence. Trial centre or region-specific *per diem* hospital costs will be based on NHS reference costs in England and cost information for NHS Scotland derived from the Scottish Health Service Costs resource. Probabilistic sensitivity analysis will also be used to assess the hospital cost distributions.

We will assess differences in costs and effects using econometric methods based on a copula framework, which is particularly useful and straightforward when modeling joint parametric distributions. We will also summarize the cost effectiveness results within a net benefit approach using incremental net (monetary) benefit and cost-effectiveness acceptability curves.

Costs and benefits of an effective approach to preventing DVT following stroke will accrue over time. An important element of the economic analysis will be a focus on longer run outcomes using a decision-analytic model that builds on the within-trial findings. The key parameters for the patient level simulation model will include expected survival, quality of life, long-term complications, such as post-thrombotic syndrome, and use of health services over a 6-month to 5-year time horizon. The model will be calibrated using distributions from reported systematic reviews of survival and health-related quality of life following stroke and the long-term prognosis, and cost burden of DVT in the community. Monte Carlo probabilistic sensitivity analysis will be used to account for uncertainty in the cost effectiveness results based on the simulation model.

### Other planned analyses

We will examine the association between baseline variables and the occurrence of post stroke DVT. We will replicate the analyses performed in the CLOTS 1 and 2 trials to verify findings in those trials [9,10]. We will also examine the strength of association between the occurrence of DVT within the first 30 days and longer-term survival and OHS.

We will use this dataset to externally validate the models previously developed by our group and used in the minimization algorithm to predict the OHS at 6 months [4]. This dataset will also be used to externally validate a statistical model that we have built in the CLOTS 1 and 2 trial datasets to predict which patients will respond to postal questionnaires at 6 months.

## Abbreviations

ADL: activities of daily living; CI: confidence interval; DMC: data monitoring committee; DVT: deep vein thrombosis; IPC: intermittent pneumatic compression; MRI: magnetic resonance imaging; OHS: Oxford handicap scale; PE: pulmonary embolism; QALY: quality adjusted life year; rtPA: recombinant tissue plasminogen activator; VTE: venous thromboembolism.

## Competing interests

The authors have no financial or non-financial interests relevant to the submitted work; except that Covidien (Mansfield, MA, USA) provide free supplies of Kendall™ SCD Express Sequential Compression System (IPC devices and sleeves) to hospitals participating in the trial. Neither Covidien or the funders of the study have any role in data collection, data storage, data analysis, drafting of reports or the decision to publish; although we will allow Covidien to comment on draft manuscripts describing the main results prior to final submission.

## Authors’ contributions

MD is the Chief investigator, obtained the funding for the trial, supervised its completion and drafted the statistical analysis plan. PS is a co-investigator, grant holder, member of the trial steering committee and commented on drafts of the statistical analysis plan. GM is a statistician, a member of the trial steering committee and commented on drafts of the statistical analysis plan. JF is co-investigator, grant holder and a health economist, a member of the trial steering committee. He wrote the Health economic analysis plan and commented on drafts of the statistical analysis plan. All authors read and approved the final manuscript.
